# Supplementary material for: The complete mitochondrial DNA of the carnivorous sponge Lycopodina hypogea is putatively complemented by microDNAs
Source: PeerJ. 2024 Nov 15;12:e18255. doi: 10.7717/peerj.18255 (PMC11572364; doi:10.7717/peerj.18255)
Supplement: Supplemental Information 1 [file peerj-12-18255-s001.pdf]

Supplementary Material S1 – PCR amplifications of arrangements from the mitochondrial genome of *Lycopodina hypogea*

In order to prove particular arrangements in the mitogenome of *Lycopodina hypogea* recovered through bioinformatics, selected regions along mitochondrial chromosomes were amplified through PCR. Specific primers were designed to amplify five fragments, three on chrM1 and one each on *putative* chrM2 and chrM3. Fragments on chrM1 comprised regions between genes commonly found adjacent in the mitogenomes of other Poecilosclerida sponges. Primers on *putative* chrM2 and chrM3 were designed in order to test if they comprise or not unique linear sequences (H_0). For such (Figure S1), forward sites were located downstream to reverse sites in order to only allow amplifications if annealing sites were to be found again either in a circular structure (H_1) or tandem sequence repeats (H_2). Primers used in this survey are presented in Table S1. Specific primer sets were expected to generate products according to the organization of mitochondrial chromosomes of *Lycopodina hypogea* (Figure 5; Table S2).

PCR amplifications were conducted in 30 µL reactions using 1.0 U GoTaq DNA polymerase (Promega, Madison, USA), in proprietary buffer, 0.13 mM of each dNTP, 0.17 µM of each primer, and ca. 100 ng of DNA template, with exception of amplifications of regions longer than 1 kbp, which were conducted in 50 µL reactions using 0.5 mM of each dNTP. Amplifications were conducted in a Veriti™ Thermal Cycler (Applied Biosystems, Foster City, USA), under conditions: for regions below 1 kbp, a denaturation step at 94 °C per 4 min, followed by 35 cycles of 94 °C per 15 s, 58 °C per 30 s, and 72 °C per 45 s, and a final extension step at 72 °C per 6 min); and for regions above 1 kbp, a denaturation step at 94 °C per 6 min, followed by 35 cycles of 94 °C per 1 min, 58 °C per 1 min, and 72 °C per 4 min, and a final extension step at 72 °C per 15 min. PCR amplifications included reactions using primers diplo-rnl-f1 and diplo-rnl-r1 (Lavrov et al., 2008) as positive control (Figure S2). PCR products were visualized under UV light after 2% agarose gel electrophoresis and ethidium bromide staining. Product sizes were estimated through linear regression between molecular weight bands from 1 Kbp Plus DNA Ladder (Invitrogen, Waltham, USA). Digestion of PCR products with *Alu*I restriction enzymes were carried in 15 µL reactions following manufacturer's conditions.

Table S1. Primers used in this study.

Primer	Sequence (5′ → 3′)	Temp (°C)	Reference
diplo-rnl-f1	TCG ACT GTT TAC CAA AAA CAT AGC	58.0	Lavrov et al., 2008
diplo-rnl-r1	AAT TCA ACA TCG AGG TSG GAA AC	58.0	Lavrov et al., 2008
M1-nd1-f29111	GTG CTC TGT ATT TTG GGG CC	58.0	This Study
M1-rnl-r1650	AGA CAG TGG GGC ATT CGT TA	58.0	This Study
M1-rns-f19472	GGC ATT AGA GAA GCG TGT GG	58.0	This Study
M1-cox1-r23499	ATT CCT GGG TGA CAA AAG CG	58.0	This Study
M1-cox3-f10733	CGG AGC AAG CAT ATC ATC CC	58.0	This Study
M1-cytb-r14041	CAT AAT TAC ACA GAC CCC GGC CT	58.0	This Study
M2-f199	GGG GAG TCG GGT ATA TGT TTG	58.0	This Study
M2-r120	AAC CGA GGC CGC CAA TAA TA	58.0	This Study
M3-f81	GCT GAT TAA CAA AGT AGG CCG T	58.0	This Study
M3-r62	GGA CTT GAA CCC ACC ACC	58.0	This Study

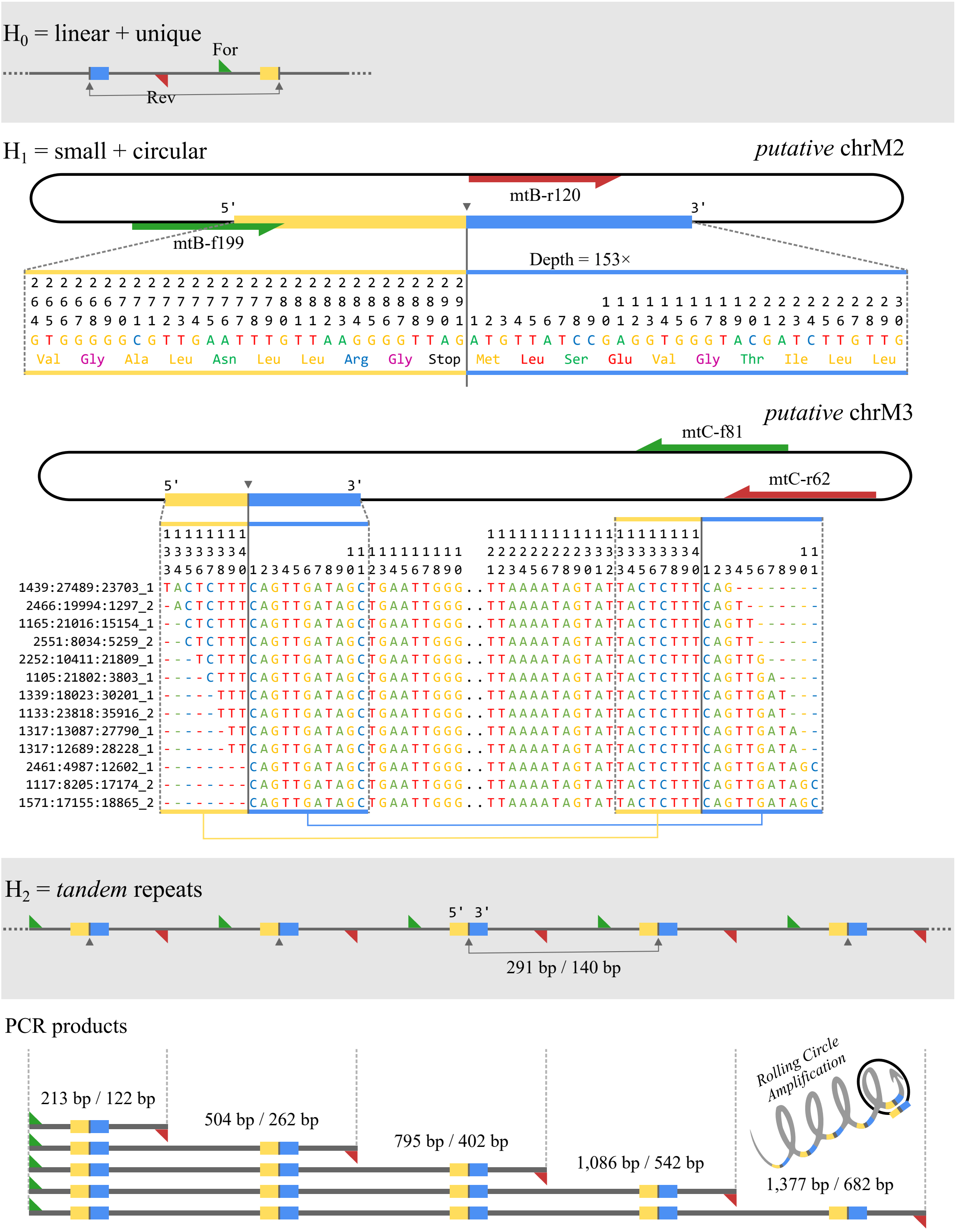

Figure S1. Three hypotheses to explain putative mitochondrial chromossomes 2 (chrM2) and 3 (chrM3) sequences on *Lycopodina hypogea* metagenome. H_0. *Putative* chrM2 and chrM3 comprise unique segments from linear molecules. H_1. *Putative* chrM2 and chrM3 comprise small, circular sequences, as presented before, able to undergo Rolling Circle Amplification. H_2. *Putative* chrM2 and chrM3 comprise tandem repeats. Expected size of PCR products produced by H_1 and H_2 for *putative* chrM2 and chrM3.

Table S2. Successful PCR amplifications and expected results using specific primer sets.

Primer set	Amplification	Obs. size (bp)	Exp. size (bp)
diplo-rnl-f1 + diplo-rnl-r1	Positive	680 [650–850]	698
M1-nd1-f29111 + M1-rnl-r1650	Negative	–	3,649
M1-rns-f19472 + M1-cox1-r23499	Negative	–	4,0258
M1-cox3-f10733 + M1-cytb-r14041	Positive	3,329 [3000–4000]	3,309
diplo-rnl-f1 + M1-rnl-r1650	Positive	219 [200-300]	221
M2-f199 + M2-r120	Positive	239 [200–300]	213
Additional fragments		525/813/1,116/1,405	504/795/1,086/1,377
Repeating unit (*putative* chrM2)		291.6 ± 8.1	291
M3-f81 + M3-r62	Positive	146 [100–200]	122
Additional fragments		285/434/568/704	262/402/542/682
Repeating unit (*putative* chrM3)		139.4 ± 6.5	140

We failed to obtain PCR amplifications for the regions between *nd1* and *rnl*, and *rns* and *cox1* genes from mitochondrial chromosome 1 (chrM1) of *L. hypogea*. Failure to amplify these regions should not be taken as evidence for absence of these arrangements; long PCR reactions are challenging without optimizations, and further experiments are needed to validate these hypotheses. However, we successfully amplified the region between *cox3* and *cytb* genes. According to our assembly, this region is expected to span 3,309 bp, and we estimated the PCR fragment to have 3,329 bp, a discrepancy lower than 0.6% that might be due to limitations of the method.

Both primer sets designed to amplify regions in *putative* chrM2 and chrM3 generated successful amplifications. As predicted from both H_1 and H_2, PCR products of different sizes were generated, larger than the initial fragment between adjacent annealing sites, and at regular intervals. (Figure S2B, lanes 2–3; Table S2). For *putative* chrM2, fragments were observed 291.6 ± 8.1 apart from each other, almost equal to the 291 bp range expected for tandem repetitions of the sequence in the assembly. Fragments amplified from *putative* chrM3 were 139.4 ± 6.5 bp apart, again, approximately equal to its expected size, 140 bp. Digestion of these products using *Alu*I restriction enzyme resulted in complete disappearance of fragments above ca. 140 bp, suggesting those fragments of different sizes comprise, as predicted, tandem repeats (Figure S2C, lane 4). However, the initial fragments of both *putative* chrM2 and chrM3 were slightly larger than expected, as shown by the difference between observed and expected sizes (Figure S2D, bottom). Both reactions generated fragments, on average, 24.4 ± 5.1 and 25.5 ± 3.9 longer than expected, respectively for chrM2 and chrM3 products. Coincidentally or not, these are similar numbers from that deviation found in the product ranging from *cox3* to *cytb*, which suggests again some sort of methodological limitation.

Visualization of the purified genomic DNA of *L. hypogea*, used for library preparation, shows two bands around 290 and 140 bp (Figure S2C, lane 2). Although degradation can be seen from the high molecular weight range as a smear, intensity and pattern of degradation (*e.g.*, absence of typical bands from cleavage during apoptosis) does not explain these fragments.

Searching for the exact sequence of the junction regions (the region that connects the 3' end to the 5' start of their sequences) of *putative* chrM2 and chrM3 within the metagenome of *Lycopodina hypogea* resulted in 153 reads/14 mates and 316 reads/107 mates (60 mer, 30 mer from each end). Individually, sequences from 5' and 3' ends

were found in higher numbers, 221 and 257 reads for *putative* chrM2, and 400 and 434 reads for *putative* chrM3, including reads shorter than 30 mer in those ends, presenting point mutations, and even a couple of cases of short adapter sequences left out from trimming. But no divergent sequences that could be indicative of a region flanking those ends was found. Statistically ($\alpha = 0.05$), not finding a single flanking region among those reads (R), it would require a significant large number of repeating units (N) in tandem from *putative* chrM2 ($N_{221} \approx 74.31$; $N_{257} \approx 86.3$) and *putative* chrM3 ($N_{400} \approx 134.0$; $N_{434} \approx 145.4$), given the formula:

$$\left(1 - \frac{1}{N}\right)^R \leq 0.05$$

In conclusion, we believe these results were able to prove some hypotheses erected from the mitochondrial genome of *L. hypogea* assembled in this study, namely: 1) the existence, in chrM1, of a region ca. 3 kbp long between *cox3* and *cytb* genes; 2) the constitution of *putative* chrM2 and chrM3 in a structure either circular (H_1) or composed of tandem repeats (H_2). Other arrangements in chrM1 remain to be experimentally proved.

Unfortunately, it was impossible to determine the nature (circular or linear) or sequence (cloning) of the short molecules found in the gDNA of *L. hypogea*. That would rely on experiments, including but not limited to Southern blotting and/or in situ hybridization, and additional funding, beyond the scope and scale of the present project. In addition, DNA purifications from fresh sponge samples would be ideal to conduct such investigations. Thus, although experiments conducted here does not properly prove if *putative* chrM2 and chrM3 comprise either i) small extrachromosomal circular DNAs (microDNAs) or ii) tandem repeats in large numbers within longer segments, the seemingly presence of molecules of similar size to them in the genomic DNA of *L. hypogea* is uncanny, suggesting the former.

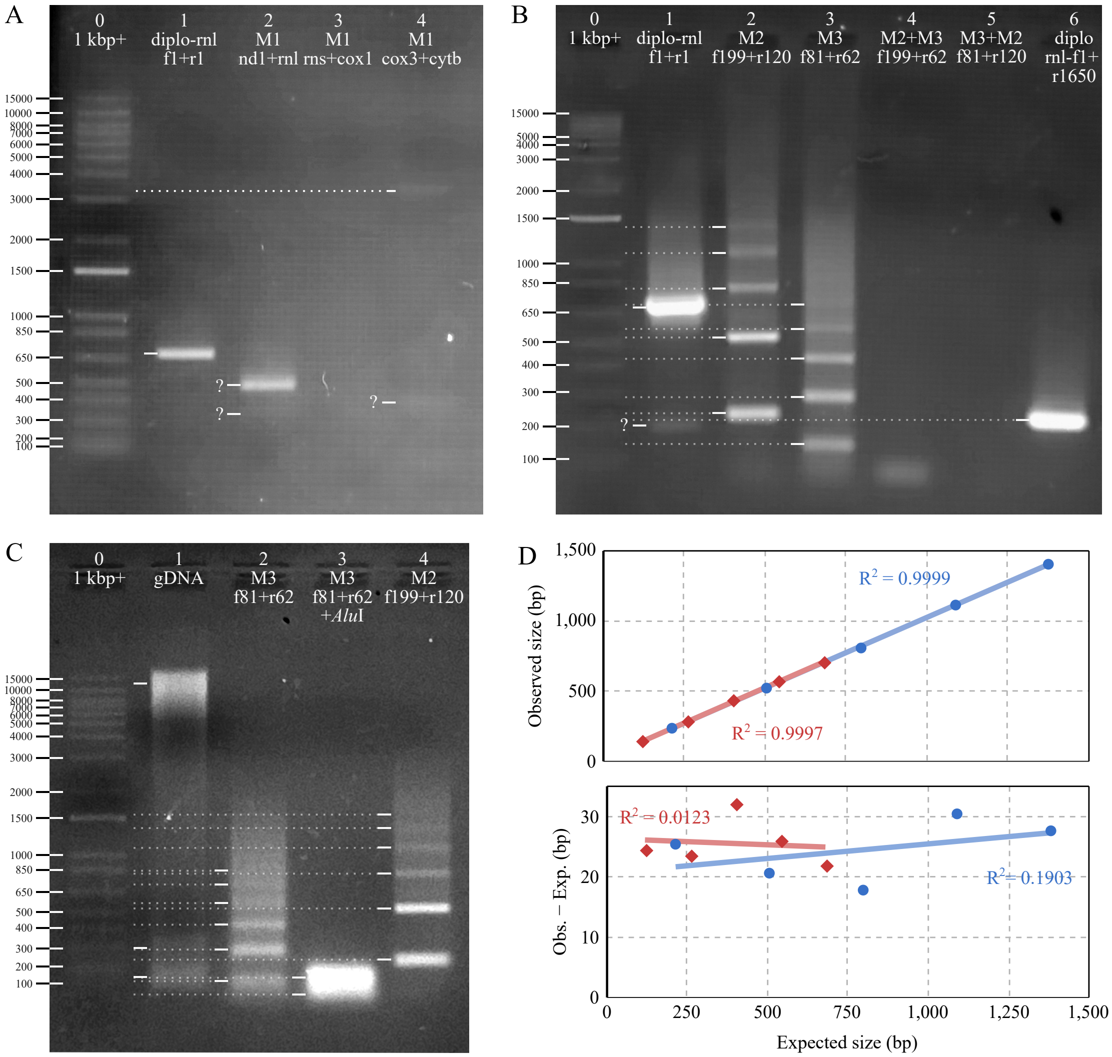

Figure S2. PCR amplifications of long (A) short (B) fragments and electrophoresis of gDNA and restriction enzyme digestion (C). Unespecific amplifications shown by a question mark (?). See Table S2 for size of fragments. C. Lane 2, high molecular weight DNA, around 14,5 kbp, and two bands at 296 bp and 139 bp; Lane 3, digestion of products from mtC-f81 + mtC-r62 amplification using *Alu*I completely cleaves tandem repeats (see lane 3), resulting in fragments of similar size, around and below 136 bp; expected restriction fragments are: 140 bp, 70 bp, and 52 bp. D. Top, Expected × observed sizes of PCR fragments, showing high coefficient of determination (R^2); Bottom, residues showing no correlation along expected fragments size.
