# Supplementary material for: The complete mitochondrial DNA of the carnivorous sponge Lycopodina hypogea is putatively complemented by microDNAs
Source: PeerJ. 2024 Nov 15;12:e18255. doi: 10.7717/peerj.18255 (PMC11572364; doi:10.7717/peerj.18255)
Supplement: Supplemental Information 2 [file peerj-12-18255-s002.pdf]

Supplementary Material S2 – BLAST searches for *putative* chrM2 and chrM3 in *Lycopodina hypogea* nuclear genome

Sequences from putative mitochondrial chromosomes 2 (*putative* chrM2) and 3 (*putative* chrM3) were searched through BLAST against the *Lycopodina hypogea* genome assembly (GCA_963969325), which was made available in the last stage of this study, which does not report extra mitochondrial chromosomes. We were able to find *nd4l* (Figure S3) and *trnI(gau)* (Table S3) partial sequences across nuclear chromosomes, which seems to comprise recombination events.

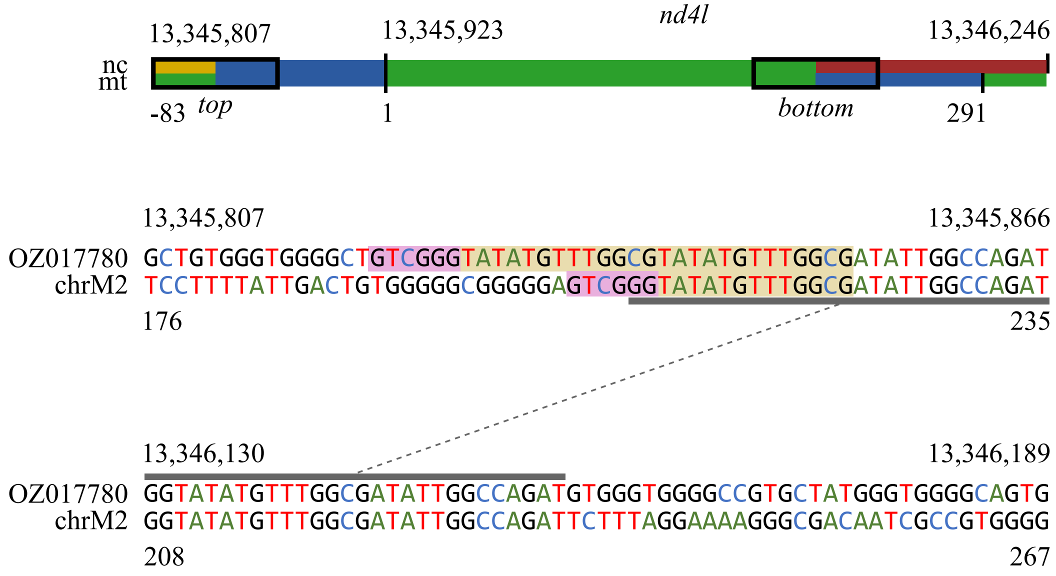

Figure S3. Comparison between *nd4l* from putative mitochondrial chromosome 2 (*putative* chrM2) and a sequence found in the nuclear genome of *Lycopodina hypogea*. Sequence OZ017780 comprises nuclear chromossome 2 of *L. hypogea*, sequences upstream (top) and downstream (bottom) putative starting codon of *nd4l* sequence found through BLAST search (OZ017780:13,345,923) showing divergent sequences, likely recombinating.

Table S3. Significant BLAST hits of putative mitochondrial chromosome 3 (*putative* chrM3) sequence, 140 bp, against the the nuclear genome of *Lycopodina hypogea* made available in the last stage of this study.

Nuclear genome	Location	Strand	Number of matches	% identity
chr2	8676206–8676288	+	83	98%
	8676315–8676353	+	39	95%
	8676289–8676319	+	31	100%
chr6	7453580–7453677	+	98	98%
chr11	2781911–2781799	-	113	95%
chr13	6656309–6656418	+	110	97%
chr14	12747081–12747215	+	135	89%
	12790618–12790478	-	140	87%
chr15	3747041–3746936	-	106	99%
