# Supplementary material for: The complete mitochondrial DNA of the carnivorous sponge Lycopodina hypogea is putatively complemented by microDNAs
Source: PeerJ. 2024 Nov 15;12:e18255. doi: 10.7717/peerj.18255 (PMC11572364; doi:10.7717/peerj.18255)
Supplement: Supplemental Information 3 [file peerj-12-18255-s003.pdf]

Supplementary Material S3 – Secondary structures of tRNAs in the mitochondrial genome of *Lycopodina hypogea*

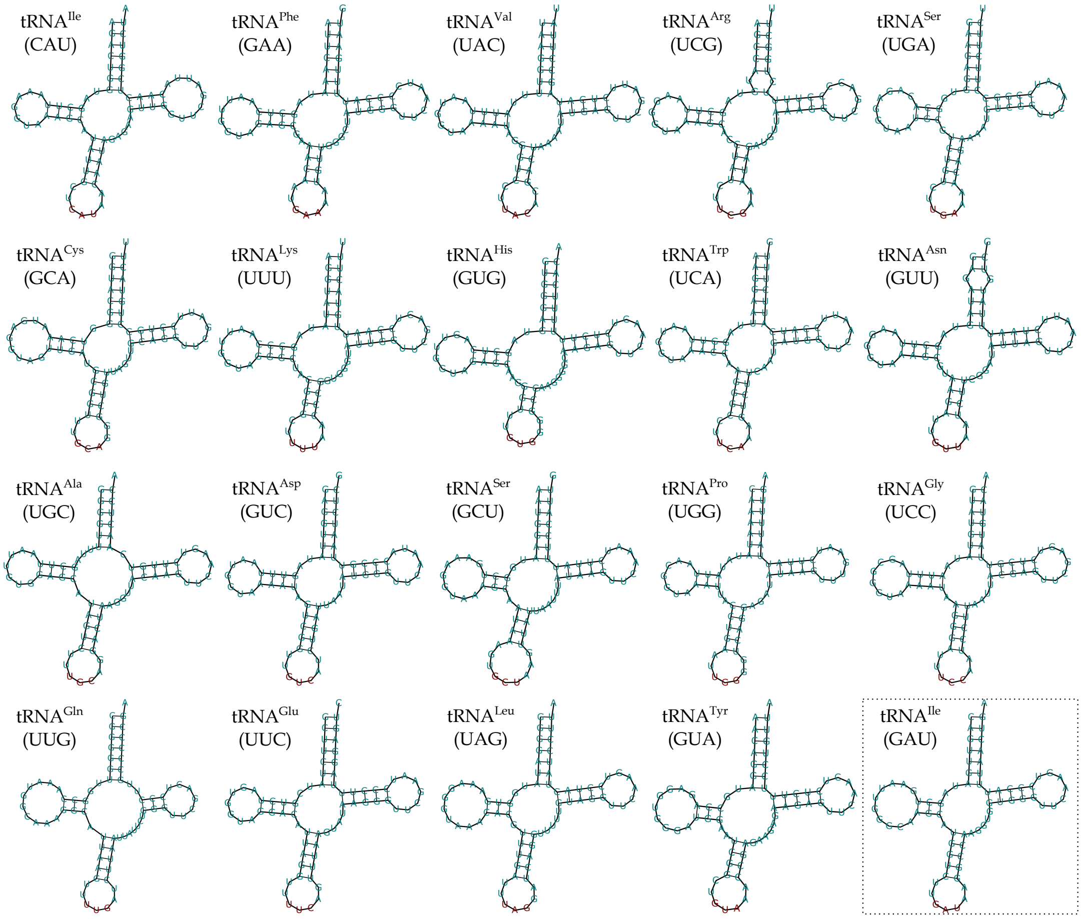

Figure S4. Transfer RNAs from the mitochondrial chromosome 1 (chrM1) and, in detail, putative mitochondrial chromosome 3 (*putative* chrM3). Anticodon sequences in red.
